# Cerebral Embolic Protection in Patients Undergoing Left Atrial Appendage Closure

**DOI:** 10.3390/jcdd12010005

**Published:** 2024-12-26

**Authors:** Julia Seeger, Philipp Seppelt, Mario Iturbe-Orbe, David Leistner, Jochen Wöhrle, Michael Joner

**Affiliations:** 1 Department of Cardiology and Intensive Care, Medical Campus Lake Constance, 88048 Friedrichshafen, Germany; 2 University of Ulm, 89081 Ulm, Germany; 3 Universitäres Herz- und Gefäßzentrum, Goethe University Frankfurt, 60590 Frankfurt am Main, Germany; 4 German Heart Centre Munich, 80636 Munich, Germany

**Keywords:** cerebral embolic protection, stroke, debris, left atrial appendage occluder, WATCHMAN FLX, SENTINEL

## Abstract

(1) Background: Cerebral magnetic resonance imaging has reported new cerebral ischemic lesions after left atrial appendage (LAA) closure in about one- third of patients. Stroke occurs predominantly periprocedurally. This study evaluated the characteristics of embolized debris captured by the SENTINEL^TM^ cerebral embolic protection system in patients undergoing LAA closure; (2) Methods: Sixty filters of 30 consecutive patients undergoing LAA closure with the WATCHMAN FLX^TM^ device were collected and captured debris was analyzed by histopathology and histomorphometry. Clinical outcome measures were disabling and non-disabling stroke within 72 h; (3) Results: In most filters, no material was captured. The predominant captured debris was acute or organized thrombi. The most common pattern was acute fibrin-rich thrombus, which was detected in 11/30 (33.3%) patients. Particles of heart tissue were seen in 6/30 (20%) patients, and foreign material was seen in one (3.3%) patient. The number of particles ranged from 0 to 52 per patient with a maximum of 31 in the distal and 21 in the proximal filter. Particle diameter ranged from 131 to 2614 µm. By logistic regression analysis, only protected time remained a multivariable predictor for larger particles (*p* = 0.039). There was no disabling or non-disabling stroke. Compared to transfemoral aortic valve replacement, the number of particles is only about 1.5%. (4) Conclusion: LAA occlusion with the WATCHMAN FLX^TM^ was associated with a very low number of embolized particles captured with the double-filter SENTINEL^TM^ embolic protection system and no periprocedural stroke.

## 1. Introduction

Percutaneous left atrial appendage (LAA) occlusion has been shown to be a safe and effective alternative to oral anticoagulation in atrial fibrillation patients at high risk of stroke and high bleeding risk [1,2]. Implantation of devices in the left atrium are known to be associated with an increased risk of cerebroembolic ischemic events, predominantly during the procedure. The left atrial appendage is anatomically diverse and characterized by a slower blood flow profile and extensive trabeculation, which both favor thrombus formation. Despite efforts in preprocedural imaging with transesophageal echocardiography (TEE), intracardiac echocardiography (ICE) and preprocedural computed tomography, small thrombi in the trabeculae might not be visualizable and risk of thrombus dislodgement and embolization is apparent. Hardly anyone uses cerebral embolic protection (CEP) for LAA closure, except in the context of isolated cases of thrombus-trapping implants featured in a handful of case reports annually. Despite the known risks of cerebral embolization during LAA procedures, limited studies have systematically analyzed embolic debris using the SENTINEL™ device.

The SENTINEL^TM^ cerebral embolic protection device, positioned via the right radial artery into the left common cerebral artery and the brachiocephalic trunk, has been shown to be a safe and effective technique, capturing debris in over 90 percent of patients undergoing transfemoral aortic valve replacement (TAVR) [3,4,5].

The aim of the present study was (1) to evaluate the safety and efficacy of the double-filter SENTINEL^TM^ embolic protection device in the setting of LAA closure, (2) to evaluate the impact of LAA closure with the WATCHMAN FLX^TM^ device on embolized debris, and (3) to compare histopathological and histomorphometric characteristics of embolized debris in the setting of TAVR and LAA closure.

## 2. Materials and Methods

### 2.1. Patients and Procedure

Consecutive patients (N = 30) undergoing LAA closure with the WATCHMAN FLX™ (Boston Scientific, Marlborough, MA, USA) were protected with the SENTINEL™ cerebral protection system (Boston Scientific, Marlborough, MA, USA). Captured debris in both filters was analyzed for histopathology and histomorphometry by the German Heart Centre Munich. Patients with a history of non-valvular atrial fibrillation and contra-indications for oral anticoagulation due to history of severe bleeding were included. Patients underwent 3D TEE the day before the procedure to assess LAA morphology and to exclude thrombus in the LAA. The SENTINEL™ cerebral protection system is a dual filter-based intraluminal embolic protection device inserted through a 6F sheath introduced via the right radial, ulnar or brachial artery. The proximal filter consists of a radiopaque nitinol frame with a 140 µm pore polyurethane filter and is positioned in the brachiocephalic trunk. The distal filter is similarly constructed and is inserted into the left common carotid artery. The two filters cover all brain areas supplied by the right vertebral and right and left carotid arteries, comprising more than 90% of the cerebral perfusion. The SENTINEL^TM^ device was inserted after establishment of femoral access and removed after successful implantation of the LAA occluder and retrieval of the transseptal sheath from the left atrium. Procedures were performed with general anesthesia and 3D TEE guidance. Sizing was performed according to 3D TEE measurements. The diameter of LAA at the landing zone and depth of LAA were measured at 0°, 45°, 90° and 135°. Unfractionated heparin was used for periprocedural anticoagulation and given after establishment of femoral venous access. Dosage was titrated to achieve a minimum activated clotting time (ACT) of 300 s. Femoral vein access was closed by use of one ProGlide^TM^ (Abbott, Santa Clara, CA, USA). Post-procedural antiplatelet therapy included dual antiplatelet therapy with acetylsalicylic acid 100 mg per day plus clopidogrel 75 mg per day for 3 months, followed by single antiplatelet therapy. The clopidogrel loading dose was given the day before LAA occluder implantation after TEE imaging.

The protocol complies with the Declaration of Helsinki and was approved by the local ethics committee. The prospective study is registered with DRKS 00022411. Written informed consent was obtained from all patients prior to any study procedure. Histopahtologic and histomorphometric analyses was performed blind to patients` clinical data. Baseline data and periprocedural data including clinical, device and echocardiographic (transthoracic and transesophageal) data were captured.

The primary outcome measure was the histophathologic and histomorphometric analysis of captured debris in both filters. The study size was limited to thirty patients by the ethics committee, since this is the first trial for consecutive patients undergoing LAA closure with the WATCHMAN FLX^TM^ device in combination with the dual-filter SENTINEL^TM^ cerebral protection system. The clinical outcome measure was clinical disabling or non-disabling stroke or transient ischemic attack within 72 h after the procedure. Stroke was defined as an acute episode of a focal or global neurologic dysfunction caused by vascular injury to the brain, spinal cord, or retina resulting from hemorrhage or infarction [6]. In patients in whom stroke was suspected, neuro-imaging had to be performed.

### 2.2. Histomorphometric and Histopathology Analyses

A total of 60 filters (30 proximal and 30 distal) from the SENTINEL™ cerebral protection system containing embolic debris captured during WATCHMAN FLX™ implantation were shipped to the German Heart Centre Munich fixed in 10% neutral buffered formalin. The collected sample material was processed in a graded series of alcohols and xylene for embedding in paraffin. Following complete hardening of the paraffin blocks, 5 μm cross sections were cut using the semi-electronic rotary microtome Rotary 3005 E from pfm medical. The generated cuts were stretched on a water bath and mounted to charged histology slides. To ensure complete adhesion of the cuts, the slides were baked at 56 °C for at least 1 h. Afterwards, slides were stained with hematoxylin and eosin (H&E) and Movat pentachrome staining. All sections were examined by light microscopy to assess the type of thrombi, and the presence/absence of endocardial/myocardial/epicardial heart tissue, atrial wall material, leaflet material and foreign material. Histomorphometric analysis was performed to quantify the number of retrieved particles, to assess the particle diameter and the cumulative particle area.

Histology slides were photographed and digitalized using either an Olympus microscope (BX 41, Olympus, Tokyo, Japan) with the associated camera (DP 74, Olympus, Tokyo, Japan) or an automated slide scanner (Aperio AT2, Leica). Morphometric measurements were taken in HE images using either cellSens Imaging Software (cellSense Standard 1.17, Olympus, Tokyo, Japan) or Aperio ImageScope (Version 12.3.3.5048, Leica Biosystems, Wetzlar, Germany).

**Figure 1:**
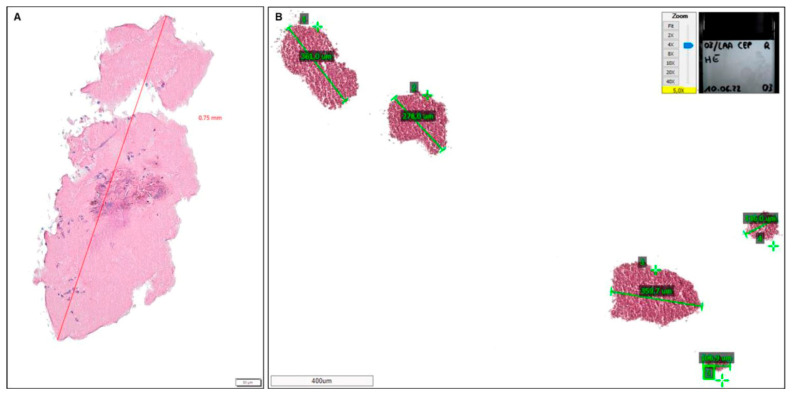
Morphometric measurements in cross-sections. The number of particles as well as the particle diameter are measured either with cellSense (**A**) or Aperio ImageScope (**B**), depending on the device used for pictures acquisition. (Example pictures show: (**A**) LAACEP 30_L, HE staining, 10× magnification, box indicates 50 µm; (**B**) LAA-CEP 03_R, HE staining, 5× magnification, box indicates 400 µm).

**Figure 2:**
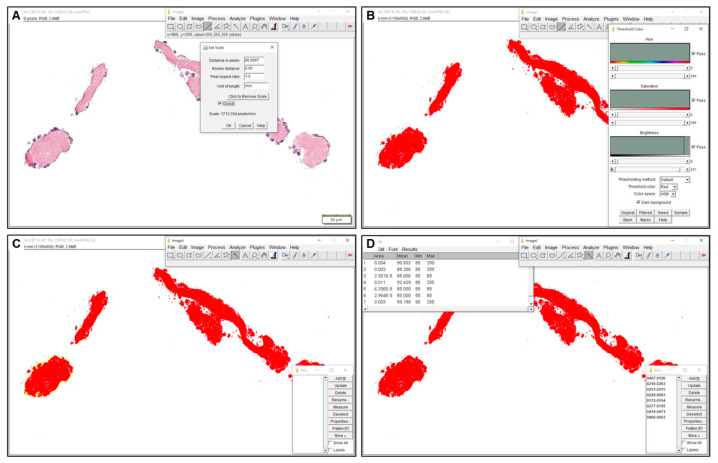
Morphometric measurements in cross-sections. The cumulative particle area was measured using ImageJ. The procedure was the same for both imaging devices. A calibrated scale was used to convert pixel into mm (**A**). Color threshold recognition was applied to automatically highlight particle size and area (**B**). By using the magic wand tool, all particle areas were added to the ROI manager (**C**) and the areas were calculated by ImageJ version 1.53 in mm^2^ (**D**). (Example pictures show: (**A**–**D**) LAA-CEP 01_R, HE staining, 10× magnification, box indicates 50 µm).

The following measurements were performed in the cross-sections: number of particles, particle diameter (μm) and cumulative particle area (mm^2^). An overview of the different measurements is detailed in Figure 1 and Figure 2.

**Figure 3:**
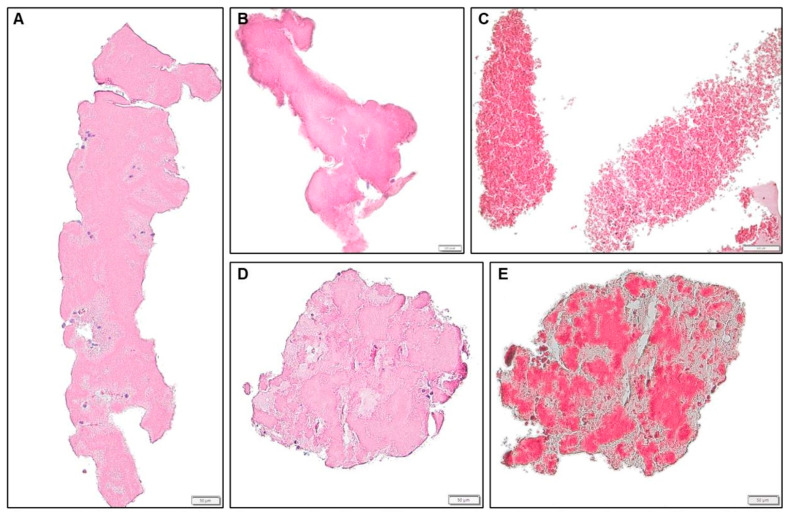
Representative images for the different sub-categories of acute thrombi, HE (**A**–**D**) and Movat (**E**) staining, 10× (**C**) and 20× (**A**,**B**,**D**,**E**) magnification. (**A**): acute platelet-rich thrombus, LAA-CEP 08_R, box indicates 50 µm. (**B**): acute fibrin-rich thrombus, LAA-CEP 10_R, box indicates 200 µm. (**C**): acute RBC-rich thrombus, LAA-CEP 18_R, box indicates 200 µm. (**D**,**E**): acute mixed thrombus, LAA-CEP 05_L, box indicates 50 µm.

**Figure 4:**
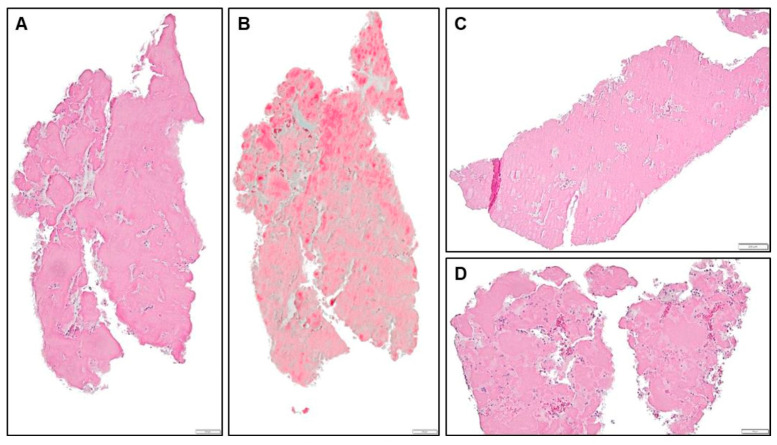
Representative images for the different sub-categories of organizing thrombi, HE (**A**,**C**,**D**) and Movat (**B**) staining, 10× (**A**,**B**,**D**) and 4× (**C**) magnification. (**A**,**B**): organizing platelet-rich thrombus, LAA-CEP 05_L, box indicates 50 µm. (**C**): organizing mixed thrombus, LAA-CEP 05_L, box indicates 50 µm. (**D**): organizing mixed thrombus, LAA-CEP 08_L, box indicates 200 µm.

Acute thrombi were assorted into four sub-categories (fibrin-rich, platelet-rich, mixed and red-blood-cell-rich), depending on their cellular composition (Figure 3). Organizing thrombi were differentiated from acute thrombi by progressive inflammatory cell infiltration and subsequent islet or lagoon formation through fibrin breakdown and digestion and assorted into three sub-categories (fibrin-rich, platelet-rich and mixed), depending on their cellular composition (Figure 4). These values and all combinations were recorded and reported for both filters separately.

### 2.3. Statistical Analysis

Categorical parameters are presented as counts and percentages and were compared by Pearson’s chi-square test and the Fisher’s exact test as appropriate. Continuous variables are presented as mean ± SD and were analyzed with ANOVA. Baseline data, procedural data and captured debris were analyzed. Univariate and multivariate predictors for capturing larger particles (>one millimeter) were analyzed. A *p* value < 0.05 was considered to be statistically significant and tests were two-sided. Statistical analysis was performed using Statistica version 10 (StatSoft, Tulsa, OK, USA).

## 3. Results

### 3.1. Baseline Data

Baseline data are detailed in Table 1. Patients had a mean age of 78 years, 50% suffered from diabetes mellitus, 30% had a history of stroke or transient ischemic attack, 10% had a history of cerebral bleeding and 70% had a history of gastrointestinal bleeding. The mean CHA_2_DS_2_VAScore and HASBLED-score were 5.5 ± 1.4 and 5.3 ± 1.2.

Both filters of the SENTINEL™ Cerebral Protection System were placed successfully in all 60 positions via right radial access. Positioning was completed in 1–2 min after sheath insertion. The protection system was in place for a mean of 37 ± 14 min. As the Sentinel^TM^ device went in before the transeptal puncture, this study nicely captures all embolic possibilities. There were no vascular complications at the radial artery. All 60 filters were shipped for histopathological and histomorphometric analysis to the German Heart Centre Munich.

### 3.2. Procedural Data

Procedural data are detailed in Table 2. The mean and median activated clotting time was 368 *s*. In three patients, more than one device was used. Repositioning for optimal device placement in order to exclude residual flow was performed in nine (30%) patients. There were no periprocedural complications such as pericardial effusion, perforation, coronary obstruction or conversion to surgery.

The diameter of LAA measured with TEE is detailed in Table 3. Oversizing of the device in relation to the LAA diameter ranged from 17.4% to 29.5% depending on the TEE view. Compression of the device ranged from 20.2% to 26.5% depending on the TEE view. There was no patient with a residual flow. Clinical (72 h) follow-up showed no event. There was no death, disabling or non-disabling stroke, TIA, hemorrhage, pericardial effusion, myocardial infarction or intracerebral bleeding. TEE after device implantation revealed all devices in situ and no device embolization, as well as no residual shunt.

### 3.3. Histomorphometry and Histopathology

Histopathologic data are detailed in Table 4 for patient level, proximal and distal filter. In most filters, no material was captured. The predominant captured debris was acute (Figure 3) or organized thrombus (Table 4, Figure 4). The most common pattern was acute fibrin-rich thrombus, which was detected in 11/30 (33.3%) patients. Particles of heart tissue were seen in six (20%) patients, and foreign material in one patient (3.3%).

**Figure 5:**
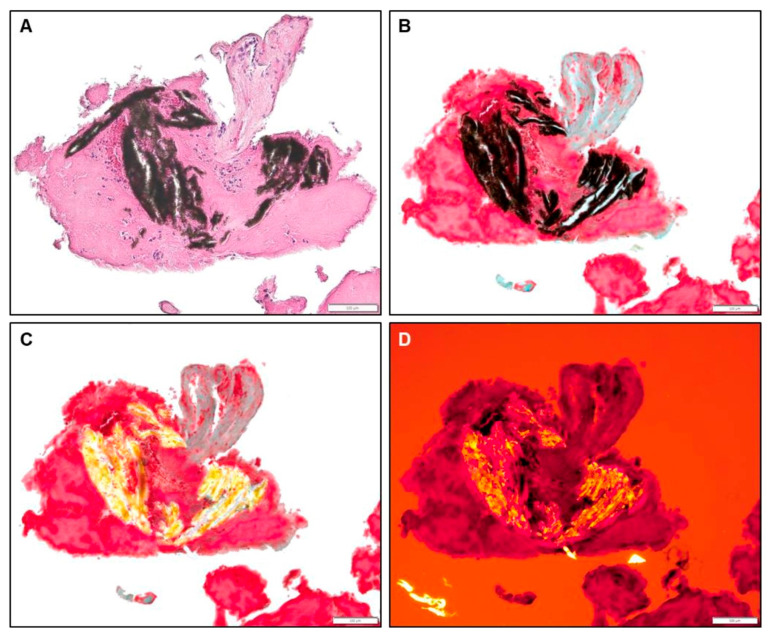
Fibrin-rich thrombus attached to endocardium and foreign material, HE (**A**) and Movat (**B**–**D**) staining, 10× magnification, LAA-CEP 10_L, box indicates 100 µm. (**A**,**B**): brightfield images. (**C**): polarized light, white balance adjustment, (**D**): polarized light, black balance adjustment.

**Figure 6:**
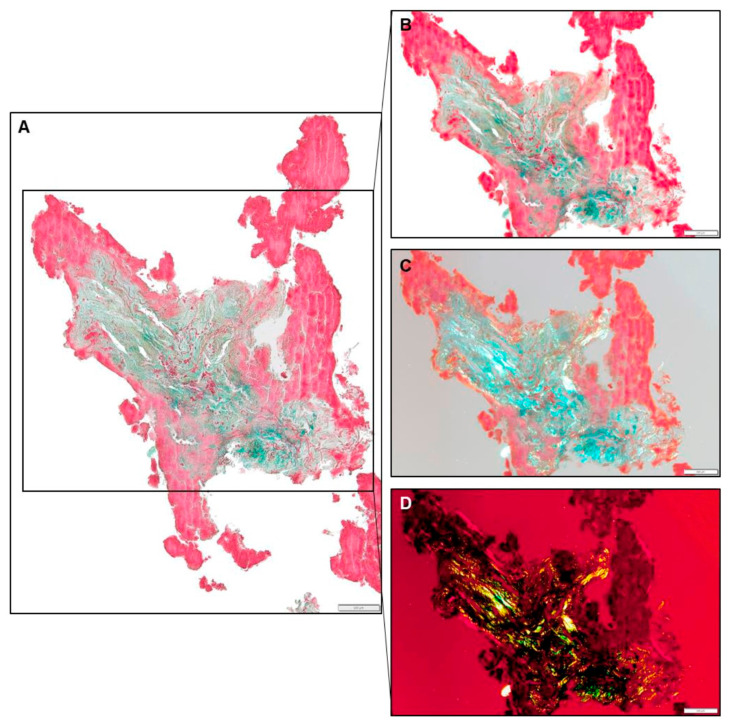
Fibrin-rich thrombus surrounding atrial wall material, Movat staining, 10× magnification, LAA-CEP 10_R, box indicates 100 µm. (**A**,**B**): brightfield images. (**C**): polarized light, white balance adjustment. (**D**): polarized light, black balance adjustment.

Figure 5 demonstrates a fibrin-rich thrombus attached to endocardium and foreign material in HE and Movat staining. Figure 6 demonstrates a fibrin-rich thrombus surrounding atrial wall material (Movat staining).

Histomorphometric data are detailed in Table 5 for the proximal and distal filter. The number of particles ranged from 0 to 52 per patient (mean 8.1 ± 11.5) with a maximum of 31 in the distal and 21 in the proximal filter. Particle diameter ranged from 131 to 2614 µm. Th mean particle diameter was 585.7 ± 393.9 µm (range 130.8–1507.0 µm) in the distal filter and 584.0 ± 541.9 µm (range 145.0–2614.0 µm) in the proximal filter (*p* = 0.992). The cumulative particle area was 3.07 ± 5.04 mm^2^ in the distal filter and 0.85 ± 1.59 mm^2^ in the proximal filter (*p* = 0.082).

In the distal (proximal) filter, 31 (16) particles larger than 1 mm were captured. Table 6 details univariate predictors for the capturing of debris larger than 1 mm. Only procedural and protected time, including the need for repositioning, were univariate predictors for particles larger than 1 mm. Protected time was 32.2 ± 7.5 min with a particle size 0–1 mm compared to 50.0 ± 18.5 min with a particle size larger 1 mm. By logistic regression analysis, only protected time remained a multivariable predictor for larger particles (*p* = 0.039).

## 4. Discussion

We show that, in patients undergoing LAA closure with the WATCHMAN FLX™, the use of the SENTINEL™ Cerebral Protection System is safe and feasible. In most filters there was no captured debris, and the number of particles was very low with a mostly single-digit number. The main histopathologic pattern was acute and organizing thrombus.

This is the first consecutive series of patients undergoing WATCHMAN FLX^TM^ implantation in combination with the SENTINEL^TM^ cerebral protection system and subsequent histopathologic and histomorphometric analysis of the captured debris. There were no complications of the protection system during implantation and placement of the device, as well as no complications at the access site. In a case series of the double-filter protection system in five patients in the context of atrial appendage occlusion, embolized material was found in the filters of the protection system in all five (100%) patients [7]. Only two of these procedures were performed with the WATCHMAN device. In addition, organizing thrombus was present in 80%, acute thrombus in 60% and tissue in 40% of these filters, supporting the apprehension that LAA closure may be similar to TAVR with respect to embolized particles. The cerebral protection system has also been used in single cases with LAA closure and thrombotic material in the LAA [8,9,10,11]. Especially in patients undergoing LAA closure with thrombus, the use of the cerebral embolic protection system may have the potential to reduce the risk for cerebral ischemic complications [12,13]. After LAA closure, the stroke rate has been reported to be up to 1.68% per 100 patient years [14]. Periprocedural stroke was a major contributor in terms of the safety endpoint, with a rate of 1.1% in the randomized PROTECT-AF trial and 0.4% in the randomized PREVAIL study [1,2]. The PINNACLE FLX study (Protection Against Embolism for Nonvalvular AF Patients: Investigational Device Evaluation of the Watchman FLX LAA Closure Technology) prospectively enrolled 400 patients undergoing LAA closure with the same device, the Watchman FLX [15]. The ischemic stroke rate after 45 days was very low, with only 0.7% (N = 3/400). A reduction in periprocedural disabling stroke with the double-filter SENTINEL^TM^ CEP device has recently been shown for TAVR in patient-level meta-analysis [16] and i n the randomized PROTECTED TAVR study [17] including 3000 patients (0.5% protected TAVR versus 1.3% unprotected TAVR, *p* = 0.02). Procedure-related stroke with LAA closure has been reported to be up to 1.1% in the PROTECT AF Clinical Trial, 0.9% in combination with the Continued Access Registry [18] and only 0.7% in the PINNACLE FLX study [15]. Periprocedural stroke rate has been significantly higher in the physician learning curve for the procedure [1,2,18]. For physicians in the learning curve for LAA closure, use of the SENTINEL^TM^ CEP system may be an option to safely treat their patients. In addition, with cerebral magnetic resonance imaging prior to and after LAA closure, new ischemic cerebral lesions have been documented in 32% of patients [19]. In the transcranial Doppler study, 116 ± 20 microembolic signals during LAA closure were detected. Since this study was performed without the double-filter CEP system, the underlying cause of microemboli material (gaseous or solid) could not be differentiated. We did not observe a disabling, non-disabling stroke, or transient ischemic attack in our series of 30 patients, which supports the good safety profile of the LAA closure device seen in the PINNACLE FLX study [15].

In contrast to TAVR, the number of particles and the captured particle area are minimal. In a large TAVR study with histomorphometric and histopathologic analysis of captured material in 200 filters, the number of particles was 172 ± 176 in the proximal filter and 176 ± 220 in the distal filter [4]. With repositioning during TAVR, the number of particles was significantly higher, with 226 ± 232 in the proximal filter and 283 ± 400 in the distal filter [4]. In contrast, in patients undergoing LAA closure with the WATCHMAN FLX^TM^ device, we found 3.6 ± 5.2 particles in the proximal filter and 4.5 ± 7.5 particles in the distal filter. The number of particles found in the same SENTINEL^TM^ CEP system after LAA closure is about only 1.5% compared to TAVR. The use of the WATCHMAN FLX^TM^ is associated with a very low risk regarding embolization of debris from the LAA during the implantation procedure. The single multivariable predictor for larger particles in the filters was the time of the protection system in place. In complex procedures with a prolonged procedure time and possible need for repeat repositioning to obtain an optimal result, the use of the CEP system may be considered.

In patients undergoing TAVR, acute thrombus captured in the filters was seen in 82.6% to 97.6% of patients depending on the implanted valve type [5]. Organizing thrombus ranged from 0% to 2.4%, valve tissue from 57.1% to 76.2%, arterial wall from 60.9% to 71.4%, calcification from 31.0% to 45.7%, foreign material from 20.0% to 21.7% and myocardium from 4.4% to 11.9%. In contrast, in the present study, including patients undergoing LAA closure, the rate of captured organized thrombus (3.3–13.3%) was higher compared to TAVR patients, whereas rate of acute thrombus (26.7–33.3%), heart tissue (20%) and foreign material (3.3%) was clearly lower, with no evidence of calcifications (0%).

It is of interest that although all patients underwent 3D TEE the day before the procedure to exclude thrombotic material in the LAA, the majority of captured debris was acute and organized thrombus. Such thrombotic material is probably located in the extensive trabeculae of the left atrial appendage, which is not clearly distinguishable from the atrial wall by TEE or other imaging methods. During LAA implantation, a second TEE study confirmed the absence of echocardiographic detectable thrombus in our consecutive series. The larger percentage of organized thrombus with LAA closure compared to TAVR is clearly explained by the thrombotic milieu in the LAA triggered by low blood flow due to atrial fibrillation and LAA enlargement. The combination of acute and organized thrombi as the main histopathologic pattern of embolized debris in LAA closure patients underscores the highly thrombotic process in the LAA. To further exclude such smaller thrombotic material in the LAA, a significant improvement of echocardiographic imaging is important.

### Limitations

This is a single-center experience. The number of analyzed filters was 60. Implantation techniques, including sizing and repositioning for optimal placement, may differ between various centers and different operators. The filter on the Sentinel^TM^ has a 140 micron mesh and therefore cannot catch debris smaller than this. We did not use intracardiac echocardiography (ICE) to exclude thrombi in the LAA. The low number of particles for LAA closure in our study has only been shown for the WATCHMAN FLX^TM^ device. Other devices may be associated with different results. The small sample size and absence of intracardiac echocardiography may limit the detection of smaller thrombi and the study’s external validity. Future multicenter studies with larger sample sizes and different cerebral protection devices are needed to validate these findings.

## 5. Conclusions

LAA occlusion with the WATCHMAN FLX^TM^ was associated with an extremely low number of embolized particles captured with the double-filter SENTINEL^TM^ embolic protection system and no periprocedural stroke.

## Figures and Tables

**Table 1:** Baseline data.

No. of Patients	30
Age, years	78.0 ± 7.0
Female, N (%)	12 (40.0%)
Body mass index (kg/m^2^)	26.2 ± 5.1
Arterial hypertension, N (%)	28 (93.3%)
Diabetes mellitus, N (%)	15 (50%)
Hyperlipidemia, N (%)	16 (53.3%)
Chronic renal failure, N (%)	12 (40%)
History of cardiac surgery, N (%)	5 (16.7%)
Atrial fibrillation, N (%)	30 (100%)
Carotid artery stenosis, N (%)	4 (13.3%)
Peripheral vascular disease, N (%)	4 (13.3%)
Coronary artery disease, N (%)	15 (50%)
History of stroke/TIA, N (%)	9 (30%)
History of cerebral bleeding, N (%)	3 (10%)
History of GI bleeding, N (%)	21 (70%)
LVEF, %	59 ± 12%
LVEF < 45%, N (%)	5 (16.7%)
Platelet count, ×109/L	220 ± 76
CHA_2_DS_2_VASc-Score	5.5 ± 1.4
HASBLED-Score	5.3 ± 1.2

Values are mean ± standard deviation (SD), LVEF = left ventricular ejection fraction, TIA = transient ischemic attack, GI = gastrointestinal.

**Table 2:** Procedural data.

No. of Patients	30
Activated clotting time, s	368 ± 70
LAA type, N (%)	30 (100%)
Chicken wing	19 (63.3%)
Cauliflower	5 (16.7%)
Windsock	4 (13.3%)
Cactus	2 (6.7%)
CEP system active, min	37 ± 14
Number of device used, N	1.2 ± 0.6
One device	27
Two devices	2
Four devices	1
Device size, mm	24.5 ± 3.5
20 mm	7
24 mm	13
27 mm	8
31 mm	1
35 mm	1
Device repositioning, N (%)	9 (30%)
One time	5
Two times	2
>two times	2
Conversion to surgery, N (%)	0 (0%)
Adjunctive PCI, N (%)	0 (0%)
Coronary obstruction, N (%)	0 (0%)
Pericardial effusion, N (%)	0 (0%)
Perforation, N (%)	0 (0%)

Values are mean ± standard deviation (SD), LAA = left atrial appendage, CEP = cerebral embolic protection, PCI = Percutaneous Coronary Intervention.

**Table 3:** Transesophageal echocardiography.

LAA diameter TEE, mm	
0°	18.2 ± 3.1
45°	17.4 ± 3.3
90°	16.7 ± 3.4
135°	17.8 ± 3.3
LAA depth TEE, mm	
0°	23.9 ± 6.1
45°	22.6 ± 5.5
90°	24.2 ± 4.8
135°	22.3 ± 6.0
LAA diameter TEE, mm	
Minimal	15.8 ± 3.0
maximal	19.1 ± 3.0
Min. oversizing, %	17.4 ± 6.0
Max. oversizing, %	29.5 ± 15.0
Min. compression, %	20.2 ± 3.1
Max. compression, %	26.5 ± 7.3

LAA = left atrial appendage, TEE = transesophageal echocardiography.

**Table 4:** Histopathologic data.

	Patient	Distal Filter	Proximal Filter	*p*-Value
Acute fibrin-rich thrombus, N (%)	11 (33.3%)	8 (26.7%)	5 (16.7%)	0.347
Acute platelet-rich thrombus, N (%)	8 (26.7%)	5 (16.7%)	6 (20%)	0.739
Acute mixed thrombus, N (%)	8 (26.7%)	6 (20%)	3 (10%)	0.278
Acute RBC thrombus, N (%)	9 (30%)	1 (3.3%)	9 (30%)	0.006
Organizing fibrin-rich thrombus, N (%)	1 (3.3%)	1 (3.3%)	0 (0%)	0.313
Organizing platelet-rich thrombus, N (%)	3 (10%)	2 (6.7%)	2 (6.7%)	1.0
Organizing mixed thrombus, N (%)	4 (13.3%)	4 (13.3%)	1 (3.3%)	0.161
Organized thrombus, N (%)	1 (3.3%)	1 (3.3%)	0 (0%)	0.313
Foreign material, N (%)	1 (3.3%)	1 (3.3%)	0 (0%)	0.313
Heart tissue, N (%)	6 (20%)	4 (13.3%)	5 (16.7%)	0.718

**Table 5:** Morphometric data.

	Distal Filter	Proximal Filter	*p*-Value
Number of particles	4.5 ± 7.5	3.6 ± 5.2	0.591
Range of particles	0–31	0–21	
Number of particles ≥150 μm	3.8 ± 6.8	3.0 ± 4.3	0.586
Number of particles 150–500 μm	1.5 ± 2.6	1.6 ± 2.1	0.913
Number of particles 500–1000 μm	1.2 ± 2.5	0.8 ± 1.3	0.516
Number of particles 1000–2000 μm	0.7 ± 1.7	0.4 ± 1.8	0.368
Number of particles ≥2000 μm	0.3 ± 1.1	0.2 ± 2.1	0.485

**Table 6:** Univariate predictors for large particles.

	Particle Size 0–1 mm	Particle Size >1 mm	*p*-Value
Female gender	9/21 (42.9%)	3/9 (33.3%)	0.626
Arterial hypertension	20/21 (95.2%)	8/9 (88.9%)	0.523
Hyperlipoproteinemia	13/21 (61.9%)	5/9 (55.6%)	0.745
History of smoking	10/21 (47.6%)	4/9 (44.4%)	0.873
History of stroke/TIA	7/21 (33.3%)	3/9 (33.3%)	1.0
History of intracranial bleeding	3/21 (14.3%)	0/9 (0%)	0.232
History of gastrointestinal bleeding	15/21 (71.4%)	8/9 (88.9%)	0.300
Left ventricular ejection fraction, %	58.3 ± 9.9	59.8 ± 18.1	0.780
Left atrial volume, mL	43.7 ± 18.0	55.0 ± 35.5	0.261
Right atrial volume, mL	31.2 ± 24.4	56.0 ± 45.2	0.098
TAPSE, mm	18.9 ± 4.7	21.6 ± 6.5	0.217
Troponin T, pg/mL	34.9 ± 20.3	46.6 ± 31.9	0.336
NT proBNP, ng/L	3385 ± 7338	1652 ± 1850	0.494
GFR, mL/min	57.8 ± 49.2	46.0 ± 17.9	0.493
ACT, s	368 ± 83	368 ± 21	0.988
CHA2DS2VaScore	5.5 ± 1.4	5.3 ± 1.3	0.736
HAS_BLED Score	5.3 ± 1.3	5.2 ± 1.0	0.818
Procedural time, min	58.2 ± 11.8	71.9 ± 13.9	0.010
Protected time, min	32.2 ± 7.5	50.0 ± 18.5	<0.0001
CEP positioning time, min	1.9 ± 0.9	1.8 ± 0.8	0.730
Repositioning (partial/complete), N	0.48 ± 0.87	3.22 ± 6.00	0.045
Oversizing min, %	18.2 ± 6.2	15.6 ± 5.1	0.266
Oversizing max, %	28.0 ± 10.1	32.9 ± 23.3	0.414
Compression min, %	19.5 ± 2.2	21.8 ± 4.4	0.069
Compression max, %	27.2 ± 7.9	24.7 ± 5.4	0.399
Occluder size, mm	24.0 ± 2.6	25.8 ± 4.9	0.815

ACT = activated clotting time, GFR = glomerular filtration rate, TAPSE = tricuspid annular plane systolic excursion, TIA = transient ischemic attack.

## Data Availability

The original contributions presented in this study are included in the article. Further inquiries can be directed to the corresponding author(s).

## References

[1] Holmes D.R., Reddy V.Y., Turi Z.G., Doshi S.K., Sievert H., Buchbinder M., Mullin C.M., Sick P., PROTECT AF Investigators (2009). Percutaneous closure of the left atrial appendage versus warfarin therapy for prevention of stroke in patients with atrial fibrillation: A randomised non-inferiority trial. Lancet.

[2] Holmes D.R., Kar S., Price M.J., Whisenant B., Sievert H., Doshi S.K., Huber K., Reddy V.Y. (2014). Prospective randomized evaluation of the Watchman Left Atrial Appendage Closure device in patients with atrial fibrillation versus long-term warfarin therapy: The PREVAIL trial. J. Am. Coll. Cardiol..

[3] Kapadia S.R., Kodali S., Makkar R., Mehran R., Lazar R.M., Zivadinov R., Dwyer M.G., Jilaihawi H., Virmani R., Anwaruddin S. (2017). Protection against cerebral embolism during transcatheter aortic valve replacement. J. Am. Coll. Cardiol..

[4] Seeger J., Romero M., Schuh C., Virmani R., Wöhrle J. (2019). Impact of repositioning during transcatheter aortic valve replacement on embolized debris. J. Invasive Cardiol..

[5] Seeger J., Virmani R., Romero M., Gonska B., Rottbauer W., Wöhrle J. (2018). Significant differences in debris captured by the Sentinel dual-filter cerebral embolic protection during transcatheter aortic valve replacement among different valve types. JACC Cardiovasc. Interv..

[6] Lansky A.J., Messé S.R., Brickman A.M., Dwyer M., van der Worp H.B., Lazar R.M., Pietras C.G., Abrams K.J., McFadden E., Petersen N.H. (2017). Proposed standardized neurological endpoints for cardiovascular clinical trials: An Academic Research Consortium initiative. J. Am. Coll. Cardiol..

[7] Meincke F., Spangenberg T., Kreidel F., Frerker C., Virmani R., Ladich E., Kuck K.H., Ghanem A. (2017). Rationale of cerebral protection devices in left atrial appendage occlusion. Catheter. Cardiovasc. Interv..

[8] Saccà S., Ferro J., Umemoto T., Turri R., Penzo C., Pacchioni A. (2017). A New strategy for transcatheter left atrial appendage closure with cerebral embolic protection in patient with left auricular thrombosis and total contraindication to long-term anticoagulation. J. Invasive Cardiol..

[9] Boccuzzi G.G., Montabone A., D’Ascenzo F., Colombo F., Ugo F., Muraglia S., De Backer O., Nombela-Franco L., Meincke F., Mazzone P. (2021). Cerebral protection in left atrial appendage closure in the presence of appendage thrombosis. Catheter. Cardiovasc. Interv..

[10] Beneduce A., Ancona F., Marzi A., Radinovic A., D’Angelo G., Agricola E., Della Bella P., Mazzone P. (2019). Percutaneous treatment of persistent left atrial appendage thrombus using Watchman FLX no-touch implantation technique and cerebral protection yystem. JACC Clin. Electrophysiol..

[11] Gaspardone A., D’Errico F., Iamele M., Piccioni F., Iani C., Sgueglia G.A. (2018). Single transseptal puncture for left atrial appendage closure and mitral valvuloplasty with total cerebrovascular protection in a patient with acute embolic cerebral ischemia. JACC Cardiovasc. Interv..

[12] Torres-Saura F., Cruz-González I., Pérez-Berbel P., Centurión-Inda E.R., Dau-Villarreal D.F., Romero-Vazquiánez M., Arroyo-Úcar E. (2020). Left atrial appendage closure with the use of a WATCHMAN FLX device in a patient with persistent left atrial appendage thrombus. Can. J. Cardiol..

[13] Tan B.E., Depta J.P. (2021). Transcatheter cerebral embolic protection during WATCHMAN procedure in two patients with persistent left atrial appendage thrombus: Case report with review of the literature. Catheter. Cardiovasc. Interv..

[14] Reddy V.Y., Doshi S.K., Kar S., Gibson D.N., Price M.J., Huber K., Horton R.P., Buchbinder M., Neuzil P., Gordon N.T. (2017). 5-year outcomes after left atrial appendage closure: From the PREVAIL and PROTECT AF trials. J. Am. Coll. Cardiol..

[15] Kar S., Doshi S.K., Sadhu A., Horton R., Osorio J., Ellis C., Stone J., Shah M., Dukkipati S.R., Adler S. (2021). Primary outcome evaluation of a next-generation left atrial appendage closure device: Results from the PINNACLE FLX trial. Circulation.

[16] Seeger J., Kapadia S.R., Kodali S., Linke A., Wöhrle J., Haussig S., Makkar R., Mehran R., Rottbauer W., Leon M. (2019). Rate of peri-procedural stroke observed with cerebral embolic protection during transcatheter aortic valve replacement: A patient-level propensity-matched analysis. Eur. Heart J..

[17] Kapadia S.R., Makkar R., Leon M., Abdel-Wahab M., Waggoner T., Massberg S., Rottbauer W., Horr S., Sondergaard L., Karha J. (2022). Cerebral embolic protection during transcatheter aortic-valve replacement. N. Engl. J. Med..

[18] Reddy V.Y., Holmes D., Doshi S.K., Neuzil P., Kar S. (2011). Safety of percutaneous left atrial appendage closure: Results from the Watchman Left Atrial Appendage System for Embolic Protection in Patients with AF (PROTECT AF) clinical trial and the Continued Access Registry. Circulation.

[19] Majunke N., Eplinius F., Gutberlet M., Moebius-Winkler S., Daehnert I., Grothoff M., Schürer S., Mangner N., Lurz P., Erbs S. (2017). Frequency and clinical course of cerebral embolism in patients undergoing transcatheter left atrial appendage closure. EuroIntervention.

